# The effects of segmentation on cognitive load, vocabulary learning and retention, and reading comprehension in a multimedia learning environment

**DOI:** 10.1186/s40359-023-01489-5

**Published:** 2024-01-02

**Authors:** Dongyang Liu

**Affiliations:** School of Foreign Languages, Henan University of Animal Husbandry and Economy, Zhengzhou, Henan 450046 China

**Keywords:** Segmentation, Multimedia environment, Vocabulary learning, Vocabulary retention, Reading comprehension, Chinese learners

## Abstract

**Background:**

Segmentation is a common pedagogical approach in multimedia learning, but its effects on cognitive processes and learning outcomes have yet to be comprehensively explored. Understanding the role of segmentation is crucial, as it has the potential to influence the way instructional materials are designed and delivered in digital learning environments.

**Objectives:**

This research aims to fill this gap by examining the impact of segmentation on cognitive load, vocabulary acquisition, retention, and reading comprehension in a multimedia learning context.

**Methodology:**

Participants were selected from two language schools in Zhengzhou through a multi-stage random sampling method. Ninety teenage students were randomly assigned to six experimental groups. The study utilized a 2 × 3 factorial design to examine segmentation and textual augmentation effects. Four assessment instruments were employed: a Reading Comprehension Test, a Vocabulary Assessment Test, a Cognitive Load Assessment Scale, and a Prior Knowledge Test. The experiment comprised four stages: pre-test, Instruction, post-test, and follow-up. Data analysis was performed using SPSS 22 software, involving descriptive statistics, one-way, and multi-way analysis of variance.

**Results:**

Results indicated that high segmentation significantly impacts cognitive load, vocabulary learning, retention, and reading comprehension across various aspects of multimedia learning. In essence, segmentation reduces cognitive load, supports learning efficiency, and facilitates more profound understanding, vocabulary learning, and retention.

**Conclusions and implications:**

High segmentation in multimedia learning significantly impacts cognitive load, vocabulary learning, comprehension, and retention. Educators should prioritize segmentation for more effective and engaging e-learning experiences.

**Supplementary Information:**

The online version contains supplementary material available at 10.1186/s40359-023-01489-5.

## Introduction

In recent years, the rapid advancement of technology has revolutionized the landscape of education, offering new and innovative opportunities for learning. One of the notable developments in this domain is the utilization of multimedia learning environments, which has garnered significant attention from researchers and educators alike [1–9]. These environments have shown great promise in enhancing the educational experience across various academic disciplines. Multimedia learning environments encompass various tools and instructional strategies, including animations, on-screen text, voice narration, and interactive components, making them a versatile platform for delivering educational content [7–9].

In this era of globalization, proficiency in the English language is increasingly recognized as a fundamental requirement for individuals across diverse academic fields and professional settings. The English language has established itself as a global lingua franca, serving as a primary means of communication for information exchange [10, 11]. Consequently, pursuing English language proficiency has become a paramount goal for many learners.

Scholars and researchers have investigated the impact of multimedia learning environments on language acquisition, specifically within the context of English language learning. These investigations have demonstrated that multimedia learning environments are pivotal in facilitating language acquisition and enhancing learning outcomes [12]. The educational landscape in the realm of English language learning is multifaceted, involving the acquisition of language skills at various levels, from basic vocabulary to advanced comprehension [12].

Vocabulary knowledge is a fundamental component of language proficiency, particularly in the context of second language learning. Learners rely heavily on their vocabulary to communicate effectively in a second language. Extensive research has emphasized the importance of acquiring a substantial vocabulary to engage in meaningful conversations and comprehend a wide range of texts [13]. Vocabulary learning is a complex process that encompasses deliberate vocabulary acquisition, where learners intentionally focus on expanding their vocabulary and incidental vocabulary learning, which occurs as a byproduct of language use without specific learning objectives [14].

In pursuing vocabulary learning, learners often engage in reading as a primary method. Reading exposes learners to a variety of contexts and contexts, enabling them to encounter new words and reinforce their understanding of previously learned vocabulary [14]. While reading is regarded as an effective tool for enhancing vocabulary knowledge, some studies have suggested its limited impact on vocabulary learning [15]. Nevertheless, the consensus among scholars is that reading contributes significantly to vocabulary acquisition [15].

With the advent of multimedia learning environments, new possibilities have emerged for enhancing vocabulary learning. Researchers have explored the potential of multimedia presentations and explanations to improve vocabulary acquisition and reading comprehension. Multimedia illustrations combine text, images, and sometimes audio to aid learners in understanding and retaining vocabulary. As such, multimedia explanations have been applied to enhance vocabulary learning, directing learners’ attention to word meanings and forms within the context of their reading material [16, 17].

The effectiveness of multimedia explanations in vocabulary learning has been a keen interest among scholars. Studies by Chan and Plas [18] demonstrated that combining text and images in multimedia explanations proved to be more effective than text alone or text paired with video explanations. The emergence of multimedia-integrated computer technology has ushered in new prospects for educators and researchers seeking to enrich the foreign language learning experience [19]. This extends to vocabulary acquisition and pedagogy, as evidenced by prior research [20–22]. Vocabulary scholars have been advancing vocabulary instruction by scrutinizing the role of multimedia input, such as multimedia glosses [20, 21]. In the context of our study, multimedia input is defined as the integration of text, audio, images, and videos for language acquisition, serving as a vital stimulus for various facets of vocabulary knowledge. It facilitates learners in establishing referential connections between two distinct mental representation systems: the verbal and the visual [22–25].

This study seeks to investigate the impact of segmentation within multimedia learning environments on learners’ cognitive load and its influence on vocabulary learning and retention. Segmentation is a crucial principle in multimedia learning that suggests presenting instructional materials in segments that align with the cognitive pace of learners. By breaking lessons into manageable chunks, segmentation offers learners control over the speed of Instruction, thereby facilitating their ability to manage cognitive processing [27–29].

In the era of advanced multimedia learning environments, it is essential to understand how these multimedia tools can be optimized to enhance vocabulary acquisition while minimizing cognitive load as it pertains to the segmentation of instructional materials. This study aims to contribute valuable insights to second language learning by investigating how the use of segmentation in multimedia environments affects vocabulary learning and retention. By exploring these principles, this research endeavors to enhance the efficiency and effectiveness of multimedia materials for language learners.

Despite the significant body of research exploring the intersection of multimedia learning environments, vocabulary acquisition, and cognitive load in the context of second language learning, there remains a notable gap in the literature. Specifically, there is a lack of in-depth investigation into the nuanced factors within segmentation strategies that may influence learners’ cognitive load and subsequently impact vocabulary learning and retention.

While existing studies acknowledge the importance of segmentation as a fundamental principle in multimedia learning, they often provide limited insights into the optimal design of segmented instructional materials for vocabulary acquisition. The literature lacks a comprehensive exploration of how varying forms of segmentation, such as the duration of segments, the nature of content division, and the pacing of information delivery, contribute differentially to learners’ cognitive load and vocabulary outcomes. Additionally, the majority of research has focused on the general impact of multimedia interventions on language learning, often neglecting the unique challenges and opportunities posed by vocabulary acquisition. A more granular examination of how segmentation within multimedia presentations specifically influences the intricate process of learning and retaining vocabulary in a second language is notably absent from the existing body of literature.

Furthermore, while some studies have touched upon the effectiveness of multimedia explanations and presentations, there is a dearth of research that systematically disentangles the role of segmentation within these multimedia contexts. This study aims to address this gap by providing a nuanced exploration of how specific aspects of segmentation, as applied in multimedia learning environments, can optimize the cognitive load for learners engaged in vocabulary acquisition. In essence, the existing literature provides a foundation for understanding the broad relationships between multimedia learning, cognitive load, and vocabulary acquisition. However, a focused investigation into the impact of segmentation strategies on these interconnections is crucial for advancing instructional design principles tailored to the intricacies of second language vocabulary learning. This research seeks to bridge this gap by offering detailed insights into the nuanced effects of segmentation within multimedia learning environments on learners’ cognitive processes and their ultimate success in acquiring and retaining a richer vocabulary in a second language.

## Literature review

Schüler, et al., [17] conducted a study investigating the impact of presenting lengthy textual content on sensory and augmentation effects in students from diverse academic disciplines. The findings of this research highlighted the significance of adhering to multimedia design principles within specific text length (short sentences) and segmentation boundaries. Learners exposed to segmented conditions experienced favorable learning outcomes. However, the study also indicated that augmentation enhances performance by reducing cognitive effort. Extensive processing of written text exhibited a broader relationship with learning outcomes. One critique of this study was its exclusive focus on students with high reading and information-processing skills. Nevertheless, the reliability of the memory and transfer tool was found to be low.

In their study, Jansharovatan et al., [30] examined the influence of sensory modality and control of presentation speed on multimedia learning among second-grade high school students. They discovered that the speech modality and learner-controlled presentation speed led to improved performance. This supported the cognitive load theory. However, the study did not consider individual differences among the learners.

Valaati et al’ s [31] research delved into cognitive load management and its effects on memory and learning of English grammar among second-grade male middle school students. The study found that reducing external cognitive load, managing internal cognitive load, or applying both strategies simultaneously increased students’ learning. Simultaneous management of internal cognitive load and reduction of external cognitive load also enhanced retention. The study did not take into account individual differences.

Hasanzadeh et al., [32] examined segmentation and redundancy in multimedia learning for fifth-grade female students. The results indicated that the speech modality outperformed text, revealing a sensory effect. However, the redundancy group, which received both text and speech, demonstrated reduced performance in the transfer test due to information redundancy. Individual differences were not considered. Hosseinzadeh et al., [33] investigated the impact of multimedia instruction based on the cognitive load theory in math classes for fifth-grade female students. Their findings indicated that this approach significantly improved learning, retention, and academic motivation. It was shown to be an effective tool for enhancing the learning process.

Liu [34] investigated the effects of intra-device multimedia principles and sensory pathways on learners who had chosen English as their second language. The study examined three input modes (text, graphics, and audio + text) regarding the augmentation principle among students who had selected English as their second language. The results indicated that different input modes did not significantly impact memory retention and vocabulary test performance among students who had chosen English as their second language. Thus, the sensory and augmentation principles were non-significant.

Mayer [26] has posited that multimedia input effectively sustains learners’ cognitive engagement, particularly when they are involved in selecting pertinent materials, structuring content into visual and verbal models, and integrating these new models with their preexisting knowledge. This sustained cognitive engagement can alleviate the cognitive burden on learners regarding their working memory. Furthermore, multimedia can assist learners in managing their intrinsic cognitive load, optimizing their germane load, and reducing their extraneous load, all of which collectively enhance information retention for more effective learning outcomes [26].

Chen and Yen [35] explored the effects of learner control, segmentation, and presentation quality in animated presentations as pre-class guidelines. Multimedia principles such as segmentation, learner control, and quality were recommended to enhance the effectiveness of animated displays. However, applying multimedia principles in various learning contexts with different learner profiles revealed boundary conditions for other effects. Therefore, this study aimed to investigate different levels of learner control, segmentation, and augmentation effects on learning and cognitive load when participants were given time to interact and learn. Two experiments, low control vs. high control, were conducted with a 2 × 2 design (segmentation: unsegmented vs. segmented, sensory pathways: narrated vs. written). A supplementary analysis in both experiments was performed to differentiate the primary effects of learner control and segmentation. The findings of this study provide essential insights for designing pre-class guidelines to enhance learner interaction and comprehension. The results of the second experiment demonstrated that written text can encourage participants to interact more with the Instruction than spoken text alone. These findings suggest that creating a narrated presentation with additional written explanations (subtitles) can benefit novices.

Ramezanali et al. [25] conducted a study to examine the effectiveness of the elaborative interrogation method concerning one versus two and two versus three types of explanations for learning new words in a second language. The study reviewed twenty-two studies and examined moderating variables such as data sample quality, learner-related variables, explanation features, text features, and cognitive method features. The results indicated that the overall effect of the elaborative interrogation method was moderate for immediate post-tests (d = 0.46) and small for delayed post-tests (d = 0.28). Mediation analysis revealed that the effects of the elaborative interrogation methods are influenced by a range of learner-related variables (e.g., skill), explanation features (e.g., language), research design (e.g., testing method), and text features (e.g., storytelling vs. explanatory). Adding one way to descriptive text resulted in increased word learning. However, adding explanation method to two explanations did not improve word learning. The results suggest that employing more than two explanatory methods is unnecessary as it does not enhance word learning.

Chan, et al., [14] examined the impact of additional on-screen text in learning from a PowerPoint presentation with animated content narrated by a native or non-native (foreign) speaker, without text, brief text, or full text. Participants completed retention and transfer tests and assessed cognitive load from narration and PowerPoint content. With a native speaker, participants without text outperformed those with brief reader on the transfer test (augmentation effect). It appeared that understanding the narration of a foreign speaker was more challenging than that of a native speaker.

### Objectives of the study

This study aims to explore the effects of segmentation on cognitive load, reading comprehension, vocabulary retention, and learning in L2 learning within a multimedia environment for Chinese EFL learners. The research seeks to address the existing gaps in the literature and provide insights into optimizing multimedia-based L2 education. In line with these objectives, the following questions were raised:

1. Does segmentation in a multimedia learning environment affect Chinese EFL learners’ cognitive load?
2. Does segmentation in a multimedia learning environment affect Chinese EFL learners’ vocabulary learning?
3. Does segmentation in a multimedia learning environment affect Chinese EFL learners’ vocabulary retention?
4. Does segmentation in a multimedia learning environment affect Chinese EFL learners’ reading comprehension?
5. Does segmentation in a multimedia learning environment have a long-term effect on Chinese EFL learners’ reading comprehension?

## Method

### Participants

Among the six language schools in Zhengzhou, a method of multi-stage random sampling was employed to select two schools for inclusion in the study. Subsequently, these two schools adopted a random selection process to identify 90 teenage students. The selected students were then arbitrarily assigned to six distinct experimental groups. Notably, the participants in this research were actively enrolled in English language education during the third and fourth terms and could be classified as moderately novice language learners.

The current study employed a fully randomized 2 × 3 factorial design, aiming to investigate the impacts of segmentation (high segmentation versus low segmentation) and textual augmentation (auditory, visual, and combined). The total sample size consisted of 90 subjects, who were randomly distributed across the six experimental groups, each comprising 15 participants.

### Instruments

#### Reading comprehension test

The reading comprehension test used in this study was adapted from the “Reading and Writing” book published by Oxford University Press. This test consisted of 16 multiple-choice questions. This test scored 0 to 16, with one point awarded for each correct answer (Appendix 1). Expert judgments were employed to establish the content and face validity of the research tests, as is customary for content validity. This study’s test content was derived from academic sources and documents (specifically, the “Reading and Writing” book published by Oxford University Press, focusing on volcanoes). To ensure content validity, the opinions of the research supervisor were sought.

Furthermore, after preparing the tests, they were reviewed by five experts in the field of English language education. Any identified shortcomings and issues were addressed, and the tests were modified accordingly. As a result, the content and face validity of the reading comprehension and vocabulary tests were assessed and confirmed. Following the determination of content and face validity, the prepared tests were administered experimentally to a group of 10 students (who were English language learners but not part of the sample group) after they received Instruction and read computer-based texts. Each question’s essential characteristics, including its difficulty level and clarity, were computed. Questions with inappropriate difficulty coefficients were removed from the tests. The difficulty level of the questions on the reading comprehension questionnaire fell between 3.0 and 7.0 on the scale, categorizing them as moderately tricky questions. Questions with difficulty levels ranging from 7.0 to 10.0 were considered manageable, and those with 0 to 3.0 were labeled very difficult. The ideal range for question difficulty is between 3.0 and 7.0.

Moreover, the discrimination index was calculated, which indicates the degree to which a test question distinguishes between high- and low-performing students. The discrimination index for the 16 questions on the reading comprehension test ranged from 4.0 to 8.0, demonstrating a relatively high discrimination index. To calculate the reliability of the test, Cronbach’s alpha was utilized. The Cronbach’s alpha obtained for this research was 0.78.

#### Vocabulary assessment test

The vocabulary test is a researcher-constructed test consisting of 16 questions. Each question in this test covers one of the target vocabularies. In this test, participants were required to choose the equivalent Persian meaning for each English vocabulary word provided in the questions. The questions in this test were divided into three categories: writing the word’s meaning, matching the word to its appropriate definition, and substituting the word in a sentence. Four vocabulary words were given for the “writing the word’s meaning” category, and six words were included in both the “matching” and “substituting” categories. In total, the vocabulary test comprised 16 questions divided into three categories (Appendix 1). Scoring for the vocabulary test ranged from 0 to 16, with one point awarded for each correct answer. The difficulty coefficient for the 16 questions on the vocabulary questionnaire ranged from 5.0 to 7.0, categorizing them as moderately tricky questions. Questions with difficulty coefficients between 7.0 and 10.0 were considered easy, while those ranging from 0 to 3.0 were labeled very difficult. The ideal range for question difficulty falls between 3.0 and 7.0.

Similarly, the discrimination index for each question was calculated to assess the degree to which the question distinguishes between high- and low-performing students. The discrimination index for the 16 questions on the vocabulary test ranged from 4.0 to 8.0, signifying a relatively high discrimination index. To calculate the reliability of the vocabulary test, Cronbach’s alpha was employed, resulting in an alpha value of 0.73 for this research.

### Cognitive load assessment scale

The Cognitive Load Assessment Scale, developed by [16], was used to measure cognitive load. The scale consists of nine levels, ranging from very low cognitive effort (1) to very high cognitive effort (9). Paas, Van Merriënboer, and Adam [17] demonstrated the reliability and sensitivity of this scale, indicating that one-dimensional scales are sensitive to cognitive load differences and are both valid and reliable.

The first question in this scale assessed the difficulty level in instructional content, using a Likert 9-degree spectrum from very, very easy to very, very difficult. Each participant rated the instructional content based on their experience with the educational program. The second question measured the extent of mental effort by participants on a Likert 9-degree spectrum, ranging from very, very low to very, very high. Participants indicated their level of mental effort expended in comprehending the instructional content.

To establish the validity of this scale, expert judgments were sought concerning the categorization of cognitive load. Two English language experts reviewed and corrected a translated questionnaire, and it received further validation from relevant professors. For reliability assessment, the questionnaire was administered to a group of 20 individuals from the population (excluding the sample group), and Cronbach’s alpha coefficient was calculated to be 0.82. Although the scale measures cognitive load with only two items, the high alpha value may be attributed to a strong correlation between these items (0.79), as reported in previous studies employing this scale [17].

### Prior knowledge test

The prior knowledge test is a researcher-constructed test designed with the assistance of five experts in the field of English language education. It consists of seven questions. Initially, six multiple-choice questions related to volcanic activity were included. In the seventh question, students were asked to write down anything they knew about volcanoes that needed to be covered in the first six questions. One point was awarded for each correct answer to the first six questions. The open-ended question at the end was worth two points. The score range for this test was from 0 to 8. A higher score indicated higher prior knowledge about the topic. Descriptive statistics, including central tendency and dispersion measures, were used for data analysis.

### Procedure

Despite the random assignment to instructional conditions, a prior knowledge test was administered to participants one hour before the experiment to ensure that the groups stayed the same in prior subject knowledge. The need for prior subject knowledge for the experiment stemmed from the fact that this topic should be equally familiar to all participants. Subsequently, participants were randomly assigned to six groups from language training classes. Two learners became test subjects in each session, and these sessions were conducted in separate computer-equipped language schools. Learners entered the class with the guidance of an English language instructor, and they randomly sat in front of a computer. In order to assess whether the participants possessed essential computer skills for participating in the experiment, they were transferred to a language laboratory before the actual test and asked questions regarding their computer familiarity. The experiment was carried out in four main stages: pre-test, Instruction, post-test, and follow-up.

The purpose of the study was explained to the participants in the pre-test stage. An informed consent form was provided, and participants were asked to read and sign it. They were informed that their participation was voluntary, their data would be anonymous and confidential, and they could withdraw their participation at any time during the study without any restrictions. Then, the participants answered prior knowledge test questions without any time constraints. A brief explanation was given to the learners about the program on the monitor screen. A scientific text on volcanoes was presented in English through PowerPoint software, consisting of 10 slides and 627 words, using Times New Roman font size 20. Each slide contained a general image describing the slide’s content and images that represented specific words. Learners could click on a word to view the corresponding image. The average duration of the experiment was approximately 15 min.

### The instructional content

The instructional content of the study encompassed a scientific explanation of volcanic activity sourced from a reading and writing book published by Oxford Publications, specifically focused on volcanoes. The material was delivered in English through the utilization of PowerPoint software, comprising ten slides (pages) and 627 words, with a font size of 20 in Times New Roman. Each slide maintained consistent image sizes and spacing between sentences. The content selection was performed collaboratively by two foreign language experts, and a software specialist arranged it within the PowerPoint software. New vocabulary words were visually highlighted in green, with multimedia presentations available for each highlighted term.

The auditory group received the presentation exclusively in an auditory format through headphones, while the visual group received the text in a visual format. Learners in the “enhanced” group received a combination of written text, images, and a spoken version of the text. To assess the segmentation effect, the low segmentation group (control system) received continuous text across two slides, each with a clear title. Learners were restricted from freely navigating between slides and experienced a 2-second pause between transitions. Conversely, the high segmentation group (self-control) encountered the selected text divided into meaningful sections across ten slides. Learners had the autonomy to navigate forward or backward using the arrow sign on each slide, including the option to return to the initial slide. Additionally, each slide featured a clear title, and learners could pause the audio playback by clicking on the stop button, allowing them to review slides at their discretion. The high segmentation group had no time constraints. In the subsequent stage, immediately following the instructional intervention, learners underwent a post-test to evaluate comprehension and vocabulary learning, along with a cognitive load test. Two weeks later, the final stage involved a vocabulary recall test, a comprehension test, and a cognitive load test, designed to assess vocabulary retention and comprehension learning.

### Data analysis methods

In this study, SPSS 22 software was used for statistical data analysis. Descriptive statistics were used to organize the data and determine central and dispersion measures, while one-way and multi-way analysis of variance was used for data analysis.

## Results

Results including descriptive statistics of the variables including Mean and Standard deviation (SD) are presented first in Table 1. Then, the results of ANOVA tests for questions are presented sequentially.

**Table 1:** Descriptive statistics of the research variables

	High segmentation						Low segmentation					
	Audio		Visual		redundancy		Audio		Visual		redundancy	
	M	SD	M	SD	M	SD	M	SD	M	SD	M	SD
Prior knowledge	7.16	0.8	7.70	1	7.30	0.80	7.2	0.8	7.7	0.80	7.56	1
Mental effort	5.30	1.2	3.46	0.7	2.53	1	5.6	0.70	4.54	0.95	5.1	1
Mental difficulty	4.7	0.70	2.73	0.6	2.26	0.70	5.5	1.3	3.75	0.90	4.65	1.1
Reading comprehension	8.30	0.90	12.1	0.90	13.2	1	7.9	0.91	10.14	1.53	9.70	1.6
Vocabulary learning	9.90	1.12	12.2	1.3	13.8	1.3	9	1.5	11.50	1.1	11.20	1

As seen in Table 1, it is evident that the “Low segmentation” condition generally leads to more favorable outcomes in terms of reading comprehension and vocabulary learning. Participants in this condition had higher scores in reading comprehension, with a mean of 12.1, and they also excelled in vocabulary learning, scoring an average of 12.2. Conversely, the “High segmentation” condition presented different challenges. Participants in this condition reported higher mental effort, with a mean score of 5.30, and perceived more mental difficulty, with a mean score of 4.7. Their reading comprehension scores were lower, averaging 8.30, and they had a vocabulary learning mean score of 9.90.

### Research question 1

The first research question investigated the effect of the segmentation method on the cognitive load of English language lessons in a multimedia learning environment. To investigate the effectiveness of the segmentation method on the cognitive load of English language lessons in a multimedia learning environment, a multivariate analysis of variance (MANOVA) was used. Results are presented in Table 2.

**Table 2:** Results of multivariate analysis of variance regarding the effectiveness of the segmentation method on cognitive load components

		SS	df	MS	F	*P*
Mental difficulty	Between groups	26.67	1	26.67	17.29	0.001
	Within groups	135.77	88	1.54		
	Total	162.45	89			
Mental effort	Between Groups	36.10	1	36.10	24.28	0.001
	Within groups	130.80	88	1.48		
	Total	166.90	89			

Results presented in Table 1 indicate a significant difference between the high and low segmentation groups in terms of mental difficulty (*P* < 0.001, F = 29.17) and mental effort (*P* < 0.001, F = 28.24), as shown in Table 1. Therefore, the segmentation method effectively reduces the cognitive load of English language lessons in a multimedia learning environment.

### Research question 2

The second research question aimed at assessing the effect of the segmentation method on vocabulary learning in English language lessons in a multimedia learning environment. To investigate the effectiveness of the segmentation method on vocabulary learning in English language lessons in a multimedia learning environment, a one-way analysis of variance (ANOVA) was used. Results are presented in Table 3.

**Table 3:** Results of one-way ANOVA regarding the effectiveness of the segmentation method on vocabulary learning

Variables		SS	df	MS	F	*P*
Mental difficulty	Between groups	26.67	1	86	5.23	0.001
	Within groups	77.135	88	4.6		
	Total	45.162	89			

Table 3 shows a significant difference between the high and low segmentation groups in vocabulary learning (*P* < 0.001, F = 66.20). Therefore, the segmentation method effectively enhances vocabulary learning in English language lessons in a multimedia learning environment.

### Research question 3

The third research question investigated the effect of the segmentation method on comprehension learning in English language lessons in a multimedia learning environment. To investigate the effectiveness of the segmentation method on comprehension learning in English language lessons in a multimedia learning environment, a one-way analysis of variance (ANOVA) was used (Table 4).

**Table 4:** Results of One-way ANOVA Regarding the Effectiveness of the Segmentation Method on Reading Comprehension

Variables		SS	df	MS	F	*P*
Mental difficulty	Between groups	*57.1*	1	56	16.29	0.001
	Within groups	*297.23*	88	3.38		
	Total	*353.29*	89			

As seen in Table 4, the results of the F-statistics in one-way ANOVA regarding the effectiveness of the segmentation method on comprehension learning indicate a significant difference between the high and low segmentation groups in comprehension learning (*P* < 0.001, F = 56.16). Therefore, the segmentation method effectively enhances comprehension learning in English language lessons in a multimedia learning environment.

### Research question 4

The fourth research question investigated the effects of the segmentation method on vocabulary retention in English language lessons in a multimedia learning environment. The results of ANOVA test are shown in Table 5.

**Table 5:** Results of one-way ANOVA regarding the effectiveness of the segmentation method on vocabulary retention

Variables		SS	df	MS	F	*P*
Mental difficulty	Between groups	*86*	1	86	17.29	0.001
	Within groups	*298*	88	3.57		
	Total	*354*	89			

As seen in Table 5, the results of one-way ANOVA indicate a significant difference between the high and low segmentation groups in vocabulary retention (*P* < 0.001, F = 27.19). Therefore, the segmentation method effectively enhances vocabulary retention in English language lessons in a multimedia learning environment.

### Research question 5

The fifth research question aimed at investigating the long-term effects of the segmentation method on comprehension in English language lessons in a multimedia learning environment. Results are presented in Table 6.

**Table 6:** Results of one-way ANOVA regarding the long-term effect of the segmentation method on comprehension retention

Variables		SS	df	MS	F
Mental difficulty	Between groups	*86*	1	85	17.29
	Within groups	*298*	88	4.42	
	Total	*354*	89		

Table 6 shows a significant difference between the high and low segmentation groups in comprehension retention (*P* < 0.001, F = 92.12). Therefore, it can be concluded that the segmentation method is effective in enhancing comprehension retention in English language lessons in a multimedia learning environment.

## Discussion

The results of the present research reveal a significant difference in the components of mental difficulty and mental effort between high-segmentation and low-segmentation groups, with the high-segmentation group reporting lower cognitive load. These findings align with previous studies by Spanjers et al. [28], Singh et al. [27], and Chen and Yen [35]. To explicate these findings, Mayer and Chandler (2001) found that students, after watching a segmented animation, can better answer transfer questions have compared to non-segmented animations (e.g., questions measuring comprehension instead of reproduction). Recent research indicates that for learners with low prior knowledge, segmentation may reduce cognitive load, leading to improved learning efficiency [27–29].

Two explanations have been proposed for the beneficial effects of segmentation on cognitive load and learning outcomes. Firstly, in previous studies on segmentation, pauses between segments have been associated. To navigate this interruption, learners engage in cognitive activities by holding information in working memory, connecting it with new, relevant information during concurrent processing [36]. When dynamic visualizations contain complex content, the external cognitive load imposed by these pauses may allow learners to perform necessary cognitive activities, preventing information loss before integration with prior knowledge [37]. Therefore, pauses between segments may have positive effects on cognitive load and learning outcomes by providing learners with sufficient time for necessary cognitive activities without simultaneous exposure to new information [2, 7, 20].

Secondly, segmenting dynamic visualizations into meaningful units may aid learning by assisting learners in grouping related elements and identifying natural boundaries between events in a process [28, 29]. People instinctively identify boundaries between events during perception, and reducing the need for this identification may reduce cognitive load, supporting learners in the process [17].

Moreover, interactive engagement with instructional materials does not appear to enhance learning compared to manipulated materials, consistent with the findings of Chi et al. [41]. They discovered that presenting text and images with lower mental effort and the need to create images with higher mental effort were associated with more positive learning outcomes. Additionally, they found more positive effects of presenting images than having learners create them. The positive effect of segmentation on mental effort is logically explained; these segments assist learners in grouping information elements that naturally belong together.

In conclusion, segmentation facilitates learning by providing learners with more time for cognitive activities necessary for learning without simultaneous exposure to new information. The external cognitive load imposed by pauses between segments may reduce cognitive load, enhancing learning efficiency. Furthermore, segmenting dynamic visualizations into meaningful units helps learners group related elements and identify natural boundaries between events, further reducing cognitive load and enhancing learning.

Furthermore, the results of this study suggest that providing learners with extra time for cognitive activities before forgetting information is essential. Thus, considering this explanation, providing additional time for learners at the end of learning does not facilitate learning. Secondly, the beneficial effects of segmentation on cognitive load and learning outcomes are likely not solely due to the presence of additional time for processing each animation. Instead, time should be allocated after each segment (e.g., a small unit of information) to enable learners to hold the information without simultaneous exposure to new input.

Lastly, segmentation with pauses between segments may benefit cognitive load and learning outcomes, providing learners with more time for necessary cognitive activities without the simultaneous presence of new information. Segmenting dynamic visualizations into meaningful units also assists learners in grouping related elements and identifying natural boundaries between events, further reducing cognitive load and enhancing learning.

### Segmentation method effect on vocabulary learning in English language lessons in a multimedia learning environment

The results of this research demonstrate a significant difference between groups with high and low levels of segmentation in vocabulary learning. The group with high-level segmentation achieved higher scores in vocabulary learning. These findings align with the results of studies conducted by [12, 13]. Our results indicate that segmentation facilitates learning and a higher degree of segmentation leads to more significant interaction with teaching and learning. This increased segmentation allows more time for cognitive processing, in line with the cognitive load theory. Repeated pauses in the learning pace allow time to process information effectively and prevent cognitive overload. Processing information too quickly, as in rapid Instruction without pauses, can hinder learning [19].

Research also shows that shorter instructional materials enhance interaction and improve learning, while more extended Instruction significantly reduces interaction [17, 28–31]. On the other hand, in high-level segmentation for learning, learners may need to engage in external processing to determine how instructions are divided, potentially resulting in improved learning scores. If longer presentations are unavoidable, learner control becomes crucial for achieving significant learning outcomes.

This research demonstrates that increased segmentation in multimedia learning environments can reduce cognitive load and promote learning and retention. Therefore, developing a comprehensive understanding of learner differentiation, including examining the degrees of segmentation, differences like learning tasks, the impact on cognitive load, the overall length of instructional videos, and learners’ prior experiences, is crucial for effective learning.

### Segmentation method effect on reading comprehension in a multimedia learning environment

The results of this research indicate a significant difference in comprehension learning between groups with high and low levels of segmentation. The group with high-level segmentation achieved higher scores in comprehension learning. These results are consistent with the findings of studies by Moreno et al. [42]. The study by Spanjers, et al. [43] (on the impact of segmented solved examples on cognitive load and learning revealed that inserting pauses between segments positively affected post-tests without affecting the invested mental effort during study. These results provide evidence for explaining the effect of segmentation through pauses, suggesting that segmentation facilitates learning because pauses give learners more time during text reading to perform the necessary cognitive processes. The positive effect of segmentation with pauses is likely not due to adding extra time for processing each text. Instead, the time should be provided after each segment (e.g., a small meaningful unit of information) to enable learners to retain information without needing new input.

Furthermore, it was found that placing pauses on each page positively impacted mental effort investment during text study: less effort was required for learning when cues were present, without affecting the success of post-learning memory. These findings provide evidence for the temporal attack explanation, suggesting that segmentation positively affects learning outcomes or invested mental effort, as it highlights natural event boundaries, reduces the need to search for those boundaries, and consequently reduces cognitive load.

### Segmentation method effect on vocabulary retention in a multimedia learning environment

The results of this research indicate a significant difference in vocabulary retention between groups with high and low levels of segmentation, with the high segmentation group achieving higher scores in vocabulary retention. These findings align with the results of studies conducted by a few researchers [17, 28, 29]. In explaining these findings, it can be said that when essential information is presented too rapidly, it can overload the learner’s cognitive capacity, leading to cognitive overload. When this happens, the learner cannot process essential information and learning outcomes effectively. A design solution used in multimedia Instruction to prevent cognitive overload is segmentation - breaking the Instruction into segments, giving learners control over the pace of Instruction through a “continue” button. Segmentation allows learners to fully process limited information sections before proceeding, enabling deeper learning [1–5]. Creating non-synchronous learning environments often involves creating self-paced multimedia instructional episodes that give learners control over the pace, rate, content, and feedback nature of learning [45–47]. Participants in high segmentation conditions have more opportunities to review words and their meanings and more time to observe them. High segmentation conditions are an effective presentation mode that allows learners to use text-based resources to a greater extent than low segmentation conditions [17, 28, 29, 35]. Therefore, learner control can reduce cognitive load, increase interaction, help learners become more specialized, and improve learning outcomes.

### Segmentation method effect on comprehension retention in a multimedia learning environment

The results of this research indicate a significant difference in comprehension retention between groups with high and low levels of segmentation, with the high segmentation group achieving higher scores in comprehension retention. These findings align with the results reported by several researchers [17, 28–42]. Doolittle et al. [47], in their investigation of the effects of segmentation degree and learner preference on multimedia learning, concluded that segmentation facilitates recall and application. Moreover, a higher degree of segmentation leads to more significant interaction with teaching and more learning.

These findings are in harmony with the theoretical assumptions of cognitive load theory, which posits that frequent pauses in the instructional pace allow time for effective information processing, preventing cognitive overload. The positive effect of segmentation with pauses is likely not just due to adding extra time for processing each text but also providing time after each segment (e.g., a small meaningful unit of information) to allow learners to process information without needing the presence of new input [25]. Interrupts, especially for tasks that require more significant cognitive needs, are crucial, and including pauses in instruction can lead to improved task performance [43]. These results also align with research related to the length of instruction. Studies indicate that shorter instructions enhance interaction and, in turn, improve learning, while more extended instructions significantly reduce interaction [42, 44–47].

## Conclusions and implications

The research on the effects of segmentation in a multimedia learning environment holds practical implications that can significantly impact instructional design and language learning practices. For instructional designers, the insights gleaned from this study offer a roadmap to optimize the design of learning materials. By incorporating segmentation strategies into multimedia presentations, educators can break down instructional content into meaningful units, fostering improved engagement and comprehension among learners.

The findings particularly benefit language educators aiming to enhance vocabulary acquisition. The positive impact of segmentation, especially when integrating pauses strategically, proves instrumental in improving vocabulary learning and retention. This understanding allows teachers to tailor multimedia resources that align with cognitive load principles, creating more effective and engaging learning materials. Therefore, educators and content developers can strategically integrate multimedia elements, such as text, images, and audio, based on the knowledge gained from this study. The combination of these elements, when segmented appropriately, enhances the overall learning experience. This insight guides the development of multimedia resources that cater to diverse learning styles and preferences, promoting a comprehensive and engaging learning environment. The study also emphasizes the importance of time management in cognitive processing during multimedia instruction. Educators can use this information to design learning activities with appropriate pauses, allowing learners to engage in necessary cognitive activities without feeling overwhelmed. This approach not only improves learning efficiency but also supports effective cognitive load management.

In adapting pedagogical practices, educators are encouraged to reconsider traditional instructional methods and explore innovative approaches that incorporate segmentation principles. This adaptation may involve training educators on effective segmentation techniques and strategies, ensuring that they are well-equipped to create impactful and engaging learning experiences for their students. Educational institutions and organizations can play a crucial role by investing in professional development opportunities for educators. This includes training on the effective integration of multimedia and segmentation techniques. By providing continuous support and updating pedagogical approaches based on the latest research findings, institutions can ensure that educators remain at the forefront of effective teaching practices, ultimately benefiting student learning outcomes. The dynamic nature of technology and education underscores the need for ongoing research in the field. Regularly updating pedagogical approaches based on the latest findings and technological advancements ensures that educators remain at the forefront of effective teaching practices, ultimately benefiting student learning outcomes.

### Limitations and suggestions for further studies

While this study sheds light on the significant benefits of segmentation in multimedia learning environments, there are certain limitations to consider. Firstly, the study primarily focused on cognitive load, vocabulary learning, comprehension learning, and vocabulary retention, neglecting potential effects on other aspects of learning. Future research could explore the broader implications of segmentation on various learning outcomes. Secondly, the study concentrated on a specific demographic, which may limit the generalizability of the findings. Future studies should consider diverse learner populations to ensure the applicability of these results across different contexts.

Additionally, the study utilized existing multimedia materials, and future research could investigate the design and development of segmented materials tailored to specific learning objectives. Furthermore, this study did not delve deeply into learners’ specific preferences and cognitive processes, which could offer valuable insights for instructional design. Lastly, the investigation focused solely on short-term outcomes, and longitudinal studies are needed to assess the long-term impact of segmentation on knowledge retention and transfer. In conclusion, while this research provides valuable insights into the advantages of segmentation in e-learning, it opens doors for further investigations to comprehensively understand its implications and explore its potential in diverse educational settings and learner groups.

### Electronic supplementary material

Below is the link to the electronic supplementary material.

Supplementary Material 1

## Data Availability

The data will be made available upon the request from the author.
