# Supplementary material for: The effects of segmentation on cognitive load, vocabulary learning and retention, and reading comprehension in a multimedia learning environment
Source: BMC Psychol. 2024 Jan 2;12:4. doi: 10.1186/s40359-023-01489-5 (PMC10759450; doi:10.1186/s40359-023-01489-5)
Supplement: Supplementary file 1 — Supplementary Material 1 [file 40359_2023_1489_MOESM1_ESM.docx]

Volcanoes are scattered across the globe, and they are most concentrated along the "Ring of Fire," encircling the Pacific Ocean. Each volcano is unique, ranging from dormant to active, and their eruptions can be awe-inspiring and, at times, destructive.

For centuries, volcanoes have captivated human interest. They serve as a window into Earth's geological processes and provide insight into the planet's inner workings. Beyond their scientific importance, they hold cultural significance in many societies. Some people worship volcanoes as deities, while others incorporate volcanic ash into their agriculture for its fertile properties.

However, these beautiful geological features also come with risks. Volcanic eruptions can disrupt ecosystems, damage property, and, in extreme cases, lead to loss of life. Understanding and monitoring volcanoes is vital for mitigating these potential dangers.

**Comprehension Questions:**

1. What is a common material expelled during a volcanic eruption?

a) Sand b) Water c) Lava, ash, and gases d) Rocks

1. How are volcanoes formed?

a) By rainfall b) By the release of gas c) By the eruption of magma d) By melting ice

1. What geological feature encircles the Pacific Ocean and has a high concentration of volcanoes?

a) The Atlantic Ocean b) The "Ring of Fire" c) The Sahara Desert d) The Himalayan Mountains

1. Why are volcanoes of interest to humans for centuries?

a) Due to their potential for causing earthquakes b) Because they are used for agriculture

c) They provide insight into Earth's inner workings and hold cultural significance

d) For their role in cooling the climate

1. What can volcanic eruptions do?

a) Improve ecosystems b) Enhance property values c) Disrupt ecosystems, damage property, and potentially lead to loss of life d) Create new land

1. What is the primary role of monitoring and understanding volcanoes?

a) To increase property value b) To worship them as deities c) To disrupt ecosystems d) To mitigate potential dangers and protect lives

1. Where is the "Ring of Fire" located?

a) Along the Atlantic Ocean b) Encircling the Arctic Circle

c) Encircling the Pacific Ocean d) In the Sahara Desert

1. What materials can be expelled during a volcanic eruption besides lava?

a) Only water vapor b) Ash, gases, and pyroclastic flows c) Ice and snow d) Rocks and dirt

**Reading Passage 2: Volcanic Hazards**

Volcanic eruptions, while awe-inspiring, can bring about various hazards, affecting both the environment and human communities. These eruptions can release not only molten lava but also ash, gases, and pyroclastic flows, which are fast-moving and searing clouds of hot gas and volcanic debris.

Ashfall, a result of volcanic eruptions, can disrupt air travel and pose risks to agriculture and infrastructure. It coats landscapes, affecting ecosystems and daily life. Volcanic gases, such as sulfur dioxide, can harm air quality and lead to health problems for those living near volcanoes. Prolonged exposure to these gases can have severe consequences for respiratory health.

Among the most perilous volcanic phenomena are pyroclastic flows, which move rapidly and are extremely hot. These deadly flows can set everything in their path ablaze, including forests and buildings, making them one of the most dangerous aspects of a volcanic eruption.

As hazardous as volcanoes can be, human communities have learned to live with them. Preparedness and early warning systems are crucial. Monitoring volcanoes, studying their activity, and establishing evacuation plans are all part of the strategy to mitigate risks.

**Comprehension Questions:**

1. What can be a consequence of volcanic eruptions?

a) Enhanced air quality b) Improved soil quality c) Ashfall and disruption of air travel d) Increased rainfall

1. What are pyroclastic flows?

a) A type of lava flow b) A type of volcanic ash c) Fast-moving and hot gas and volcanic debris clouds d) Slow-moving and cold gas clouds

1. How can ashfall impact agriculture and infrastructure?

a) It enhances crop growth and strengthens infrastructure b) It disrupts air travel and poses risks to agriculture and infrastructure c) It improves transportation and infrastructure d) It reduces water quality

1. How do volcanic gases like sulfur dioxide affect human health?

a) They have no impact on human health b) They improve air quality and respiratory health c) They have no impact on air quality d) They can harm air quality and lead to health problems

1. What makes pyroclastic flows especially dangerous?

a) They are cold and slow-moving b) They only contain water vapor c) They move rapidly and are extremely hot d) They contain only rocks and dirt

1. What can pyroclastic flows do when they travel through a forest? a) Decrease fire risk b) Improve soil quality c) Set everything in their path on fire d) Enhance plant growth
2. How do human communities prepare for and reduce the risks associated with volcanic eruptions?

a) By ignoring potential hazards b) By relocating to volcanic regions c) By implementing evacuation plans, monitoring volcanic activity, and being prepared d) By encouraging tourism in volcanic areas

1. What is one of the most dangerous aspects of a volcanic eruption?

a) Melting ice b) Pyroclastic flows c) Volcanic ash d) Rainfall and lightning

Vocabulary test

Volcanoes are scattered across the globe, and they are most concentrated along the "Ring of Fire," encircling the Pacific Ocean. Each volcano is unique, ranging from dormant to active, and their eruptions can be awe-inspiring and, at times, destructive.

For centuries, volcanoes have captivated human interest. They serve as a window into Earth's geological processes and provide insight into the planet's inner workings. Beyond their scientific importance, they hold cultural significance in many societies. Some people worship volcanoes as deities, while others incorporate volcanic ash into their agriculture for its fertile properties.

However, these beautiful geological features also come with risks. Volcanic eruptions can disrupt ecosystems, damage property, and, in extreme cases, lead to loss of life. Understanding and monitoring volcanoes is vital for mitigating these potential dangers.

1. The word "scattered" in the passage means:

a) Gathered closely b) Spread out or distributed c) Active and dangerous d) Hidden or concealed

1. In the context of the passage, "concentrated" most likely means:

a) Gathered closely or in a dense manner b) Spread thinly over a large area c) Dormant and inactive d) Hidden from view

1. What does the term "dormant" as used in the passage mean?

a) Active and erupting b) Asleep or unconscious c) Inactive, not currently erupting or showing signs of activity d) Dangerous and unpredictable

1. Based on the passage, what does "awe-inspiring" suggest?

a) Filling with a feeling of reverence, admiration, or wonder b) Causing fear and panic c) Being ordinary and unremarkable d) Lacking any visual impact

1. "Cultural significance" as mentioned in the passage refers to:

a) The geological importance of volcanoes b) The scientific value of volcanic research c) The religious or societal importance of a subject within a culture d) The physical properties of volcanic ash

1. In the passage, what is the meaning of "deities"

? a) Natural disasters b) Volcanic eruptions c) Gods or divine beings often worshiped in a religious context d) Scientific researchers

1. What is meant by "agriculture" in the passage?

a) The practice of cultivating the land, growing crops, and raising livestock b) The process of studying volcanic eruptions c) The worship of natural phenomena d) The use of volcanic ash in construction

1. The word "mitigating" in the passage implies:

a) Causing harm or destruction b) Increasing the frequency of volcanic eruptions c) Taking actions to reduce or lessen the severity of risks or dangers d) Ignoring potential hazards

Volcanic eruptions, while awe-inspiring, can bring about various hazards, affecting both the environment and human communities. These eruptions can release not only molten lava but also ash, gases, and pyroclastic flows, which are fast-moving and searing clouds of hot gas and volcanic debris.

Ashfall, a result of volcanic eruptions, can disrupt air travel and pose risks to agriculture and infrastructure. It coats landscapes, affecting ecosystems and daily life. Volcanic gases, such as sulfur dioxide, can harm air quality and lead to health problems for those living near volcanoes. Prolonged exposure to these gases can have severe consequences for respiratory health.

Among the most perilous volcanic phenomena are pyroclastic flows, which move rapidly and are extremely hot. These deadly flows can set everything in their path ablaze, including forests and buildings, making them one of the most dangerous aspects of a volcanic eruption.

As hazardous as volcanoes can be, human communities have learned to live with them. Preparedness and early warning systems are crucial. Monitoring volcanoes, studying their activity, and establishing evacuation plans are all part of the strategy to mitigate risks.

1. The word "awe-inspiring" in the passage most likely means:

a) Frightening and terrifying b) Causing wonder and admiration c) Unremarkable and dull d) Predictable and calm

1. What is the meaning of "hazard" as described in the passage?

a) A lack of danger b) Something that poses a risk or danger c) A beautiful landscape d) An environmental benefit

1. What does "searing" suggest when describing "pyroclastic flows" in the passage?

a) Cold and soothing b) Burning and scorching c) Slow-moving and harmless d) Dense and heavy

1. The term "ashfall," as mentioned in the passage, is associated with:

a) Enhancing air quality b) Disrupting air travel and posing risks to agriculture and infrastructure c) Boosting agriculture d) Creating fertile soil

1. In the passage, what does "volatile" mean when referring to volcanic gases like sulfur dioxide?

a) Unpredictable and stable b) Slow-moving and harmless c) Explosive and unstable d) Beneficial and fertile

1. What is the primary goal of "preparedness" as mentioned in the passage?

a) To promote tourism in volcanic areas b) To encourage volcanic eruptions c) To mitigate risks and reduce the impact of disasters d) To worship volcanoes as deities

1. What is the purpose of "early warning systems" as stated in the passage?

a) To attract tourists to volcanic areas b) To study the geological features of volcanoes c) To provide advance notice of potential volcanic activity d) To increase air pollution in the region

1. What is meant by "mitigate" in the phrase "mitigate risks"?

a) To increase the severity of risks b) To take actions to reduce or lessen the severity of risks c) To ignore potential dangers d) To encourage dangerous activities
